# 

**Published:** 1860-12

**Authors:** 


Transactions of the Illinois State Medical Society .... 750

Rush Medical College .... 751

Sectionalism .... 752

Remarkable Controversy .... 752

Medical Department, Lind University .... 752

### SELECTED.

Extracts from an Essay on Diptheria and Diptheritic Affections .... 709

Periodicity as a Character of Disease .... 721

Treatment of Diptheria .... 721

The Use of Instruments in Obstetric Practice .... 726

Carelessness on the part of Correspondents .... 728

Removal of the Tongue .... 728

### RECEIPTS FOR THE JOURNAL FOR NOV. AND DEC., 1860.

| | | |
|---|---|---|
| O. D. Coleman, Wis. | Vol. 3 | 2 00 |
| J. F. Weeks, Ill | " 3 | 2 00 |
| C. H. Rawson, Iowa | ½2–½3 | 2 00 |
| J. W. Lyttle, Ind. | " 3 | 2 00 |
| John Emery, Ill. | " 3 | 2 00 |
| B. J. Dunn, Ill. | ½3–½4 | 2 00 |
| D. T. Kyner, Ill | " 3 | 2 00 |
| W. G. Kittell, Ill | " 3 | 2 00 |
| L. W. Lake, Ill | " 2–3 | 4 00 |
| A. J. Minick, Ind | " 1–2–3 | 6 00 |
| Clark & Rice, Wis | " 3 | 2 00 |
| Andrew Foster, Mich. | " 3 | 2 00 |
| J. C. Helm, Ind | " 2–½3 | 3 00 |
| S. Grant Armstrong, Wis. | " 3 | 2 00 |
| John Walker, Ill. | " 3 | 2 00 |
| J. N. Denny, Ill. | " 3 | 2 00 |
| J. W. Liffingwell, Ill | " 3 | 2 00 |
| J. Tripp, Mich. | " 3 | 2 00 |
| M. M. Eaton, Ill. | " 4 | 2 00 |
| G. W. Albin, Ill | " 3 | 2 00 |
| J. P. Ross, Ill | " 3 | 2 00 |
| Thomas Winston, Ill. | Vol. 3 | 2 00 |
| James Berry, Ind. | " ½2–3–4 | 5 00 |
| A. O. Miller, Ind. | " 3 | 2 00 |
| G. O. Long, Ill. | " 4 | 2 00 |
| Wm. Carrigan, Wis. | " 3 | 2 00 |
| Dr. Mergler, Ill | " 3 | 2 00 |
| A. H. May, Mo | " 3 | 2 00 |
| Dr. Van Nus, Iowa. | " 3 | 2 00 |
| S. H. Bristol, Mich. | " 3 | 2 00 |
| J. H. Wilkinson, Ill | " 3 | 2 00 |
| E. Nichols, Ill | " 3 | 2 00 |
| J. Lamborn, Ind | " ½3–½4 | 2 00 |
| D. D. Thompson, Ill. | " 3 | 2 00 |
| S. B. W. Johnson, Wis. | " 3 | 2 00 |
| Robert Winton, Ind. | " 3 | 2 00 |
| R. A. Cameron, Ind | " 1–2–3 | 6 00 |
| C. M. Chalfant, Mo. | " 3 | 2 00 |
| W. Vanneys, Ind. | " 3 | 2 00 |
| James Pratt, Wis. | " 3 | 2 00 |
| T. W. Floyd, Ill. | " 2 | 2 00 |

### INDEX TO VOLUME III.

Administration of Medicine to Children .......... 110
After-Birth, Delivery of .......... 162
American Medical Association .......... 246
Auricles act by Contractility and Elasticity, and not by Muscular Fibre .. 428
Atropin in Epilepsy .......... 440
Ages of Pregnant Women .......... 504
Artificial Production of Bone by Transplantation of Perlosteum .......... 602

BRAINARD, DANIEL, M. D., Professor of Surgery in Rush Medical College.
Case of Lymphatic Tumor-Varix of the Lymphatic Trunk .......... 1
Dislocation of Hip into Thyroid Foramen, Reduced .......... 55
Silver Sutures .......... 55
Treatment of Indolent Ulcers by Vapors of Iodine .......... 56
Resection of Lower Jaw and removal of Parotid and Submaxillary Glands .......... 61
Amputation at the Shoulder Joint .......... 114
Amputation of Thigh for Disease of Knee Joint .......... 116
Subcutaneous Perforation of Bone in Anchylosis of the Knee Joint .. 117
Resection of the Lower Jaw .......... 215
Essay on Erectile Tumors .......... 325
Percy on Reduction of Dislocations by Manipulation .......... 408
Spina Bifida Treated by Iodine Iujections .......... 450
Notes Relating to the Extirpation of Parotid Glands .......... 569

Bright's Disease .......... 105
Bismuth in Gleet and Leucorrhea .......... 431
Bismuth in Burns and Scalds .......... 478

BOOK AND PAMPHLETS NOTICES.
F. H. Hamilton, M. D., on Fractures and Dislocations .......... 170
J. M. Carnochan on Surgery and Surgical Pathology .......... 174
Alfred Stille on Therapeutics and Materia Medica .......... 175
J. R. and J. B. Beck on Medical Jurisprudence .......... 177
Transactions of American Medical Association .......... 178
G. B. Wood, M. D., Introductory Lectures .......... 182
Pamphlet Notices .......... 169–181
New Medical Journals .......... 245
John Elwell, M. D., on Malpractice and Medical Evidence .......... 137
Prof. J. Taft on Operative Dentistry .......... 137
W. W. Gerhard, M. D., on Diseases of the Chest .......... 139
R. Bently Todd on Acute Diseases .......... 272
James Dixon, F. R. C. S., on Diseases of tho Eye .......... 277
Althans on Medical Electricity .......... 279
Charles West, Lectures on Diseases of Infancy and Childhood .......... 359
Joseph Toynbee, F. R. S., on Diseases of the Ear .......... 401
Walsh on Diseases of the Lungs .......... 553
Alfred Garrett, M. D., on Electro-Physiology and Electro-Therapeutics 557
New Medical Journel .......... 561
Henry Hartshorn, M. D., Memoranda Medica .......... 619
Druitt's Surgery .......... 621
Churchill's Midwifery ..........
Rezin Thompson, M. D., Treatise on Fevers .......... 623
Ashton on Diseases, Injuries and Malformation of the Rectum and Anus 641
O. W. Holmes, M. D., Currents and Counter-Currents in Medical Scienee 641
Forbes Winslow, M. D., on Obscure Diseases of the Brain and Disorders of the Mind .......... 745
Joseph Leidy, M. D,, Elementary Treatise on Anatomy .......... 746
M. W Hilles' Pocket Anatomist .......... 746

Chloroform and Aconite in Neuralgia.... 107
Catheterism of Air Passages. By Horace Green, M. D. 140
Chloroform in Obstetrics. 168
Chilblains. 245
Chapter of Accidents. By D. Prince, M. D. 249
Chlorate of Potash in Mercurial Salivation. 385
Cerebral Symptons, Independent of Cerebral Diseases. By Charles West, M. D. 288
Care of the Sick. 358
City Hospital, Report of. By G. K. Amerman, M. D. 377, 441
Cases of Gun-shot Wound, and Poisoning by Laudanum. By A. Groesbeck, M. D 398
Coal Tar as a Disinfectant, Velpeau. 426
Chloroform Vapor in Earache. 453
Chicago Zouaves. 503
Cancer of Mamma. Operation by T. Wilkins, M. D. 505
Chloroform, Death from. 613
Cancrum Oris. By Chas. Brackett, M. D. 638
Carelessness on the part of Correspondents 728

Diphtheria. By G. W. Dyas, M. D. 5, 68
Dislocation of Femur, 39 days' standing, Reduced by Manipulation. 54
Diphtheria, Case of. By J. J. Morgan, M D. 137
Do Bad Smells Cause Disease. 243
Death-bed of an Anatomist. 566
Diphtheria. By A. K. Van Horn, M. D. 596
Dissection, a New Method of. 614
Diphtheria and Diphtheritic Affections. 661, 709
Diphtheria. By R. B. Ray, M. D. 704
Diphtheria, Treatment of. 724

Excerpts from Medical Press. By J. A. A. 80
Extract from Gov. Wise's Speech. 165
Eye and Ear Infirmary, Report of. 356
Epidemic Disease among Cattle. 370
Effects of Disease upon Teeth. By A. Robertson, D. D. S, M. D. 519, 472,
Experimental Pathology. By Claude Bernard. 487
Experimental Pathology. 537
Experimental Pathology. 611

Fisher's Suit with H. O. Stone. 280
Fœtal Auscultation. By Francis Adams, M. D. 290
Funis Presentation. 299
Fontanelles, Closure of. 304
Fatality of Disease in London now and in the Seventeenth Century. 304
Fracture of the Ulna. By James H. Farris, M. D. 313
Florence Nightingale. 374
Femur, Gradual Dislocation of, from Disease. By H. D. Adams, M. D. 394
Fœtal Auscultation. 438
Functions of the Skin in Tubercular Consumption. 486

Glycerine Ointment for Itch. 109
Gonorrhea and Gleet Treated without Copaiba. 421
Gazette and Dr. Holmes. 750

Hemorrhage from Tonsil. 78
Hemorrhage, Nasal. 78
Hernia, Strangulated Treatment of. 185
Hysteria. By J. P. Ross, M. D. 260
Hernia, Operation for. By F. B. Haller, M. D. 393
Hypophosphates of Soda and Lime in the Treatment of Phthisis. 741

Hospital for Nervous Diseases.................................. 477
Hospital, City, Brooklyn...................................... 498
Hospital, Bellevue............................................ 548

Iodide of Potassium as an Antigalactic........................ 112
Intermittent Fever, Periodicity of. By D. S. Robinson, M. D.... ...... 134
Iodine, Tincture of, to remove Corns.......................... 283
Iodized Glycerine in Skin Diseases............................ 432
Iodism, Discussion on, before the French Academy.............. 466
Iodide of Potassium in Diseases of the Brain in Children...... 524
Ipecac instead of Tartar Emetic in Croup...................... 543
Introduction of the Tea Plant into the United States.......... 616
Iodine, Experiments with. By B. Woodward, M. D................ 697
Iodine, Cases successfully Treated with Tincture of. By E. Nichols..... 701
Introductory Address to the Class of Rush Medical College. By Prof. J. V. Z. Blaney.... 729

Lunatics in New York City..................................... 50
Letter from "Medicus"......................................... 248
Loss Sustained by Armies...................................... 440
Laryngoscope.................................................. 468
Lunatics in the Good Old Times................................ 493
Lupus, Treatment of........................................... 689
Local Effects of General Disease.............................. 691

Mutilation of a Child by a Midwife. By H. Carpenter, M. D..... 48
Mercurial Stomatitis.......................................... 52
Mystical Medical Politics..................................... 113
Medical Schools, Changes in................................... 376
Medical College of South Carolina............................. 376
Mania a Potu.................................................. 413
Medical Students and Graduates, 1855–60....................... 430
Medical Students in Paris..................................... 440
Morning Sickness.............................................. 479
Maternal Influence on Fœtal Deformity......................... 504
Mental Peculiarities and Diseases of Chilhood................. 513
Muriate of Ammonia in Nervous Cephalalgia..................... 551
Medical Education, its Tendencies............................. 681
Medical Department, Lind University........................... 752

MEDICAL SOCIETIES.
Illinois State Medical Society................................ 247, 567
Illinois State Medical Society, Meeting of.................... 279
Illinois State Medical Society, Tenth Annual Meeting of....... 360
Notice from Committees of State Societies of Indiana and Illinois .... 459
Annual Meeting of the Iowa Medical Society.................... 311
American Medical Association.................................. 409, 460
Proceedings of Dewitt County Medical Society.................. 282, 457 747
Proceedings of Rock River Union Medical Society............... 454
Proceedings of Stark County Medical Society................... 564
Proceedings of Chicago Academy of Medical Sciences............ 92, 222
April and July Meetings of the Hancock County Medical Society..... 562

Needle for Wire Suture. By R. J. Lewis, M. D.................. 51
National Society of Surgery................................... 183
Nævus Cured by Creasote....................................... 476
Nourishment in Typhoid Fever.................................. 544
Nervous Agency in Inflammatory Action. By L. S. Ellis, A. M., M. D... 585

Operations for the Restoration of the Nose.................... 25
**Obstructed Labor from Exostosis. By G. W. Phillips, M. D.** ........... 57

Opium, its Influences in Febrile, Inflammatory, and Other Diseases. By Prof. A. S. Hudson, M. D. .... 27
Opium, the uses of. By Chas. Brackett, M. D. .... 127
Opium, the uses of. By J. Blount, M. D. .... 209
Opium, an Antidote for Belladonna .... 395
Observations on Revaccination .... 527
Ointment for Warts .... 376

Physiology of the Circulation .... 16
Professional Fees .... 95
Premature Labor, Induction of .... 111
Pregnancy, Extra Uterine. By C. Goodbrake, M. D. .... 121
Physiological Incest. By W. Bird Powell .... 196
Physiological Incest. J. J. M. Angier .... 271
Parotid Gland, Abscess of- By Lykins, M. D. .... 297
Physiological Incest .... 410
Physiognomy of the Battle Field .... 433
Poisoning by Arsenic .... 439
Position in Parturition .... 494
Parotid Gland, Extirpation of .... 529
Parotid Gland, Inflammation of. By Charles Brackett, M. D. .... 640
Periodicity as a Character of Disease .... 721

Rush Medical College .... 183, 556, 751
Removal of Body of John Hunter .... 280
Rush Medical College Graduating Class, 1860 .... 281
Resection of the Entire Radius .... 504
Reed, J. H. & Co. .... 567
Remarkable Controversy .... 752

Shaft of Tibula, Removal of. By E. S. Cooper, A. M., M. D. .... 22
Southern Students leaving Philadelphia .... 53
Sulphurous Baths in Chronic Rheumatism .... 160
Stomatitis Materna. By L. L. Ellis, M. D. .... 200
Sulphurous Vapor Baths in Rheumatism. By G. L. Purdy, M. D .... 295
Sulphate of Quinine. By R. C. Hammill, M. D .... 318
Spinal Irritation. By C. D. Henton, M. D. .... 324
Saylor, Orange. Obituary .... 411
Swallowing Artificial Teeth .... 477
Sympathetic Ophthalmitis and Chronic Iritis. By E. S. Holmes, M. D. 508, 581
Sun Stroke .... 546
Subscribers in Arrears .... 563
Suture Needle, new .... 629
Spina Bifida. By John Low, M. D. .... 633
Suicide by Opium and Strychnine. Reported by J. G Pashley, M. D. .... 635
Sectionalism .... 752

To the Medical Profession .... 54
Tumor, Case of. By T. B. Cox, M. D. .... 130
Tape Worms. By E. C. Du Puy .... 213
Typhoid Fever. By A. .... 246
Tumor Intercranial. By W. J. Moody, M. D .... 385
Trowbridge, G. T., to the Medical Profession of Illinois .... 400
Tannin an Antidote to Strychnine .... 481
Training of Pugilists .... 484
Tetanus, Clinical Remarks on. .... 684
Tetanus, Case of from very slight injury By D. W. and. M. D. .... 706
Tongue, Removal of .... 728
Transactions of the Illinois State Medical Society .... 750

Uterus, Inversion of, Reduced .... 108

Ulcers, Indolent Treatment of. By B. Woodward, M. D. . . . . . . . . . . . . . . . 132
Uterus, Chronic Inversion of. . . . . . . . . . . . . . . . . . . . . . . . . . . . . . . . . . . . . 166
Union by First Intention and Purulent Absorption. By W. Stone, M. D. 284
Urethra, Stricture of. By G. Wilkins, M. D. . . . . . . . . . . . . . . . . . . . . . . . . 389
Uterine Tumor, Autopsy. By R. F. Adams, M. D. . . . . . . . . . . . . . . . . . . . . 395
Use of Instruments in Obstetric Practice. . . . . . . . . . . . . . . . . . . . . . . . . . . 725

Vesico-Vaginal Fistula. By Paul F. Eve, M. D. . . . . . . . . . . . . . . . . . . . . . 102
Variola and Vaccina. . . . . . . . . . . . . . . . . . . . . . . . . . . . . . . . . . . . . . . . . . 225
Vaccination Cases at Westford, Mass. . . . . . . . . . . . . . . . . . . . . . . . . . . . . 302
Vulvitis. By Dr. Simpson. . . . . . . . . . . . . . . . . . . . . . . . . . . . . . . . . . . . . . 305
Vitality of Toads. . . . . . . . . . . . . . . . . . . . . . . . . . . . . . . . . . . . . . . . . . . . 601

Wound of the Stomach. By James Cody, M. D. . . . . . . . . . . . . . . . . . . . . . . 264
Whooping Cough Treated with Dilute Nitric Acid. . . . . . . . . . . . . . . . . . . . 424

Zinc in Chorea. . . . . . . . . . . . . . . . . . . . . . . . . . . . . . . . . . . . . . . . . . . . . . 429

